# Polystyrene Nanomicroplastics Aggravate Ammonia-Induced Neurotoxic Effects in Zebrafish Embryos

**DOI:** 10.3390/toxics12120853

**Published:** 2024-11-26

**Authors:** Dan Xing, Wenting Zheng, Huiming Zhou, Guangyu Li, Yan Li, Jingwen Jia, Haoling Liu, Ning Luan, Xiaolin Liu

**Affiliations:** 1 College of Fisheries, Huazhong Agricultural University, Wuhan 430070, China; dan.xing@ceic.com (D.X.); zheng_teiko@hotmail.com (W.Z.); liguangyu@mail.hzau.edu.cn (G.L.); m15623588901@163.com (Y.L.); 13720270799@163.com (J.J.); liuhaoling@webmail.hzau.edu.cn (H.L.); luanning@webmail.hzau.edu.cn (N.L.); 2 CHN Energy Dadu River Hydropower Development Co., Ltd., Chengdu 610000, China; 3 Jiangxi Fisheries Research Institute, Nanchang 330039, China; mhz_507@webmail.hzau.edu.cn

**Keywords:** zebrafish, ammonia, polystyrene nanomicroplastics, neurotoxicity

## Abstract

The highly hazardous chemical ammonia has been proven to be absorbed by nanoparticles, thereby exerting highly toxic effects on aquatic organisms. As a ubiquitous pollutant in aquatic environments, polystyrene nanomicroplastics (PSNPs) have shown strong adsorption capacity due to their large surface area. Therefore, the potential joint effects of ammonia and PSNPs need to be clarified. In this study, zebrafish embryos were exposed to a water solution with ammonia concentrations (0, 0.1, 1, and 10 mg/L) with or without PSNP (100 μg/L) treatment up to 120 hpf. The results showed that combined exposure increased the accumulation of ammonia and obviously reduced the locomotor speed of zebrafish larvae compared with exposure to ammonia alone. Further studies indicated that PSNPs can aggravate ammonia-induced neurotoxicity by altering the cholinergic system, dopaminergic neurons, and the retinal structure in zebrafish larvae. In addition, our results revealed that ammonia caused significant alterations in the expression of genes related to neurodevelopment and retinal development, and PSNPs exacerbated this adverse effect. In conclusion, PSNPs can aggravate ammonia-induced neurotoxicity in the early stage of zebrafish and their associated health risk to aquatic animals should not be underestimated. The main contribution of this article lies in revealing the synergistic neurotoxicity of ammonia and PSNPs in the early stage of zebrafish. Moreover; it emphasizes that the associated health risks to aquatic animals should not be underestimated.

## 1. Introduction

Ammonia is an important pollutant in water, which mainly comes from agricultural waste, animal husbandry excrement, and industrial waste water [1,2]. Ionic ammonia (NH_4_^+^) and non-ionic ammonia (NH_3_) are the two main forms of ammonia in the water environment. NH_3_ is toxic to aquatic organisms due to its lipid solubility, which allows it to cross cell membranes freely [3,4]. Consequently, the hazards presented by ammonia to aquatic organisms should be of widespread concern. Ammonia levels in water bodies are already high due to global warming, increased intensive farming at high densities, etc. For instances, ammonia can reach 4–6 mg/L in some high-density aquaculture waters [5]. Tests at 29 water quality monitoring sites on the Liaohe River in China found that ammonia concentrations in winter could reach up to 8.12 mg/L [6].

The influence of ammonia pollution on aquatic organisms has aroused widespread attention. It has been found that ammonia has neurotoxic effects on aquatic organisms, including fish, mollusks, and crustaceans [7,8]. NH_3_ is able to penetrate the blood–brain barrier and enter aquatic organisms’ nervous system when they are exposed to high concentrations of ammonia nitrogen [9]. NH_3_ enters the nervous system and interacts with ion channels within nerve cells, leading to depolarization of the nerve cells and disturbing the normal ionic homeostasis of the cells [8], which may lead to aberrant expression of neurodevelopment-related genes, abnormal neurotransmitter metabolism, and mitochondrial dysfunction, which in turn may trigger neurotoxicity in aquatic organisms [2,10]. Most of these current studies on ammonia neurotoxicity in fish have focused on adult fish. However, studies on embryonic neurotoxicity are extremely limited.

Nanomicroplastics (NPs) are plastic particles with a particle size ranging from 1 nm to 1000 nm, mainly from industrial production, domestic waste, cosmetics, and detergents [11,12]. However, NPs enter the aquatic ecological environment due to improper handling, causing increasingly serious environmental problems [13,14]. Due to the large surface area and various forms of NPs, they can adsorb a variety of environmental pollutants, such as heavy metals and persistent organic pollutants, which can attach to the surface or interior of NPs to form complexes [15,16,17]. After NPs adsorb pollutants, they may change the chemical properties and bioavailability of the pollutants, enhancing the toxicity of the pollutants. Studies have found that the presence of polystyrene nanomicroplastics (PSNPs) increases the bioavailability of bisphenol A [18]. Zuo found that combined exposure to PSNPs and microcystin-LR increased the parental transfer of microcystin-LR to the offspring and exacerbated the growth inhibition of F1 larvae [19]. At present, studies on the co-toxicity of NPs and other pollutants have focused mainly on NPs with organic pollutants and heavy metals. Few studies have explored the co-toxicity of NPs with inorganic pollutants. It has been clearly demonstrated that ammonia can be adsorbed by nanomaterials and that both exhibit synergistic effects on the toxicity of aquatic organisms [20]. Consequently, the interaction between ammonia and NPs in the environment may lead to joint toxicity.

Zebrafish (*Danio rerio*) is widely used as a model organism in embryonic neurotoxicity experiments for toxicity evaluation [21]. The development and structure of the nervous system of the zebrafish has many similarities to that of higher vertebrates, including the formation of neurula and the migration and differentiation of neurons [22,23]. On those premises, we selected zebrafish embryos as the study subjects and exposed them to ammonia solutions (0, 0.1, 1, and 10 mg/L) without or with PSNPs (100 μg/L) for 120 hpf. The purpose of this study was to explore the combined toxic effects and mechanisms of ammonia and PSNPs on the nervous system. This study contributes to revealing the mechanism of the toxic effects of ammonia and PSNPs on the embryonic nervous system of aquatic organisms, further assessing their ecological and environmental risks, which can also be used to predict the responses of higher organisms.

## 2. Materials and Methods

### 2.1. Chemicals

NH_4_Cl (purity ≥ 99.5%) was purchased from Sinopharm Chemical Reagent Co., Ltd. (Shanghai, China). Green fluorescently labeled 100 nm PSNPs (488 nm excitation and 518 nm emission) were purchased from Tianjin Beystrat Chromatographic Technology Development Center (Tianjin, China). The other chemicals used in the experiment were analytical reagents.

### 2.2. Characterization of PSNPs Suspension

The PSNP stock solution (10 mg/mL) was added to ultrapure water to prepare fresh test solutions, which were vortexed and sonicated for 20 min. The particle size distribution of PSNPs in water particles was observed using transmission electron microscopy (TEM, HT-7700, Hitachi, Ltd., Tokyo, Japan). The particle size and zeta potential of PSNPs were characterized using a Zetasizer instrument (Malvern, UK).

### 2.3. Adsorption Experiment

To evaluate the adsorption capacity of PSNPs on NH_4_Cl, NH_4_Cl (10 mg/L) and PSNPs (100 μg/L) were mixed in a conical flask and shaken (140 r/min) for 24 h at room temperature (28 °C). Water samples were taken after 1, 2, 4, 8, 12, 16, 20, and 24 h and passed through a 0.22 μm filter membrane to remove PSNP particles. The concentration of NH_4_Cl in the water samples was determined by Nessler’s reagent spectrophotometry.

### 2.4. Experimental Design

Zebrafish embryos (AB strain) were purchased from the Institute of Hydrobiology, Chinese Academy of Sciences (Wuhan, China). At 0.5–1.0 h post-fertilization (hpf), normally developing embryos (without malformations) were selected and randomly divided across 250 mL glass beakers (200 embryos per beaker), which were later transferred to Petri dishes containing distilled water under one of the following four different conditions: control (without NH_4_Cl and PSNPs), containing NH_4_Cl (0.1, 1, and 10 mg/L), containing PSNPs only (100 μg/L), or containing a combination of the above concentrations of PSNPs and NH_4_Cl. Continuous exposure was maintained for 120 h under a light–dark cycle of 14:10 and a water temperature of 28 °C. At 120 hpf, larvae were collected on ice, quickly frozen in liquid nitrogen, and kept frozen in an ultra-low-temperature freezer (−80 °C) for subsequent analysis. Acute endpoints including hatching rate, mortality rate, malformation rate, and heart rate (heart/10 s) were recorded with at least three replicates. Embryos/larvae with no heartbeat were defined as dead and those with pericardial cysts and spinal curvature were defined as having malformation. To evaluate the heart rate, the number of heartbeats of each larvae at 72 hpf within every 10 s interval was counted. All of the above were observed under a Nikon stereomicroscope (SMZ25). In order to maintain the stability of the concentrations of ammonia and PSNPs, the experimental solution was renewed daily at the corresponding concentration and dead larvae were removed to avoid bacterial contamination of the glass beakers. The study was approved by the Institutional Animal and Care and Use Committee of Huazhong Agricultural University (IACUC, Wuhan, China).

### 2.5. Locomotor Behavior Measurement of Zebrafish Larvae

The zebrafish larvae’s locomotor behavior was quantified at 120 hpf using a video tracking system (ViewPoint LifeSciences, Montreal, QC, Canada). The larvae were placed in a 24-well plate with 2 mL purified water per well. In order to eliminate the effect of 24-well plates on zebrafish locomotor behavior, we used eight plates to measure locomotor behavior. Zebrafish locomotor behavior was monitored under continuous visible light and in response to dark-to-light transitions (5 min light, 5 min dark, 5 min light, 5 min dark). Data were collected every 30 s, including distance traveled, average speed, and movement time during dark and light periods. Each experiment was repeated four times. The data were further analyzed using custom Open Office Org 2.4 software.

### 2.6. Ammonia Content in Zebrafish Larvae

Ammonia content in zebrafish larvae was assayed using an ammonia content determination kit purchased from Addison Biotechnology Co., Ltd. (Beijing, China). Specifically, zebrafish larvae (100 per tube, n = 3) and ammonia extract were mixed at a ratio of 1:9 (*w*/*v*) and homogenized on ice for 5 min. The mixture was centrifuged at 12,000 rpm for 10 min at room temperature to obtain supernatants from all treatment groups (n = 3). The supernatant was assayed for ammonia content according to the manufacturer’s instructions.

### 2.7. Pathological Observation

Zebrafish larvae (n = 8) from each exposure group at 120 hpf were randomly selected and fixed with 4% paraformaldehyde at room temperature overnight. Afterwards, zebrafish larvae were eluted using an ethanol gradient, embedded in paraffin, and sectioned. The sections were subjected to gradient dewaxing and rehydration using ethanol. Finally, the sections were stained with hematoxylin/eosin. Histologic changes in the retina of zebrafish larvae in each exposure group were observed under an orthogonal microscope (Olympus BX53, WAKENYAKU Co., Ltd., Kyoto, Japan).

### 2.8. Gene Expression

According to the previous research method, zebrafish larvae were placed in enzyme-free centrifuge tubes (30 per tube, n = 3) and the larval tissues were cleaved by the Trizol method to extract total RNA. Then, the purity of RNA was examined by selecting RNAs with A260/A280 between 1.8 and 2.1. The cDNA was obtained by reverse transcription of pure RNA using a reverse transcription kit (TaKaRa, PrimeScript@, San Jose, CA, USA). Primer sequences for genes associated with neurodevelopment and retinal development were designed using NCBI (https://www.ncbi.nlm.nih.gov/ (accessed on 1 June 2024)). Sequences of the primers are shown in Table 1. The relative Ct method (2^−ΔΔCt^) was used to determine the relative expression of mRNA.

### 2.9. Analysis of DA Content and AChE Activity

DA content was assayed using an ELISA method developed by Nanjing Jiancheng Institute of Bioengineering (Nanjing, China). Specifically, three hundred larvae per group (n = 3) were randomly collected, and the samples were weighed and added to 9 times the volume of PBS (pH = 7.4) at a ratio of 1:9 (*w*/*v*). The samples were homogenized well with a homogenizer and then centrifuged at 3000 r/min for 20 min at 4 °C. The supernatant was collected and the DA content was determined according to the manufacturer’s instructions.

One hundred larvae from each group (n = 3) were randomly selected for the determination of AChE activity by adding 9 times the volume of saline at a ratio of 1:9 (*w*/*v*) on ice. The samples were then centrifuged at 3000 r/min for 10 min at 4 °C. The supernatant was transferred to a new tube and used for the determination of protein content and AChE activity. The kits for total protein and content and AChE activity assays were purchased from Nanjing Jianjian Bioengineering Institute (Nanjing, China).

### 2.10. Statistical Analysis

Data were analyzed using SPSS 27.0 and expressed as mean ± standard error (SEM). Data were analyzed for normality and homogeneity of variance using the Kolmogorov–Smirnov test and the Levene test. Significant differences between the control group and each exposure group were determined by one-way analysis of variance (ANOVA). Post hoc least significant difference tests were then performed using SPSS 27.0. *p* < 0.05 was considered significant.

## 3. Results

### 3.1. Characterization of PSNPs

**Figure 1:**
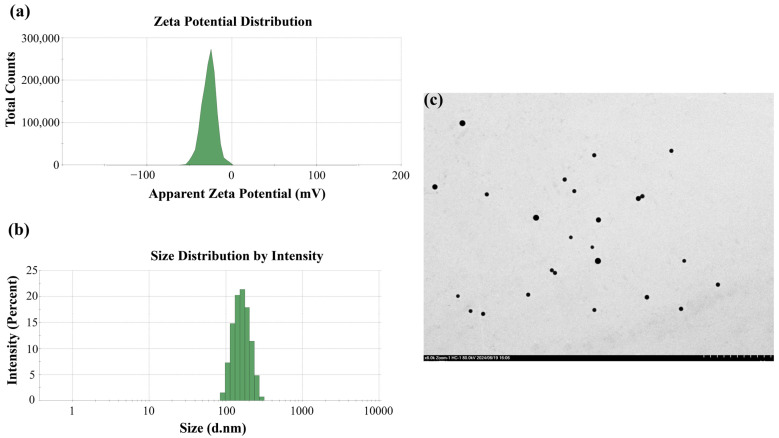
(**a**) Particle size distribution of suspension of PSNPs; (**b**) zeta potential of PSNPs; (**c**) TEM image of PSNPs. Scale bars, 200 μm.

Dynamic light scattering was used to determine the size distribution and average diameter of the PSNPs (Figure 1a); the average diameter of the PSNPs was 183.4 nm. The zeta potential of the PSNPs was −27.1 mV (Figure 1b). The particle size distribution of the PSNPs was observed using TEM (Figure 1c and Figure S1), which showed that the distribution of PSNPs was more uniform in distilled water.

### 3.2. Adsorption of Ammonia on PSNPs

**Figure 2:**
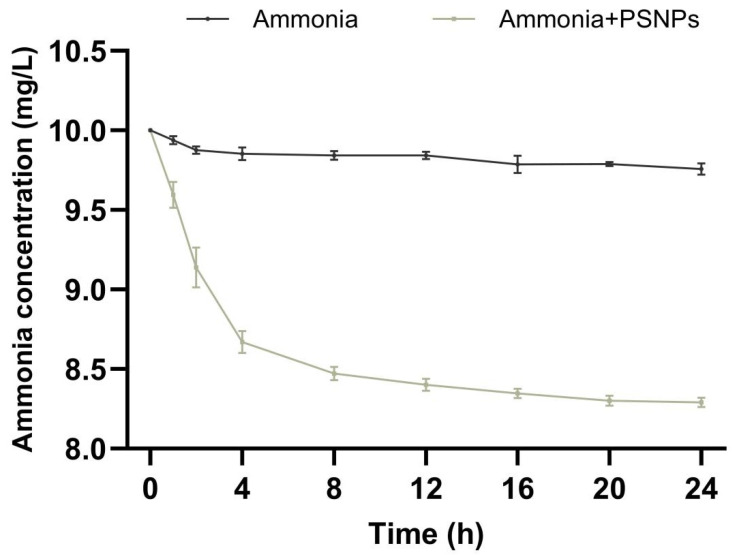
Adsorption analysis of ammonia (10 mg/L) by PSNPs (100 μg/L). The data show the changes in ammonia concentration over 24 h for ammonia alone and ammonia combined with PSNPs.

The concentration changes of ammonia (10 mg/L) alone as well as combined with PSNPs were measured over 24 h (Figure 2). The results showed that the concentration of ammonia alone changed minimally over 24 h and was relatively stable in ultrapure water. However, the ammonia concentration decreased by 17.1% from 10 mg/L to 8.29 mg/L after combining ammonia with PSNPs.

### 3.3. Developmental Toxicity of Zebrafish Larvae

**Figure 3:**
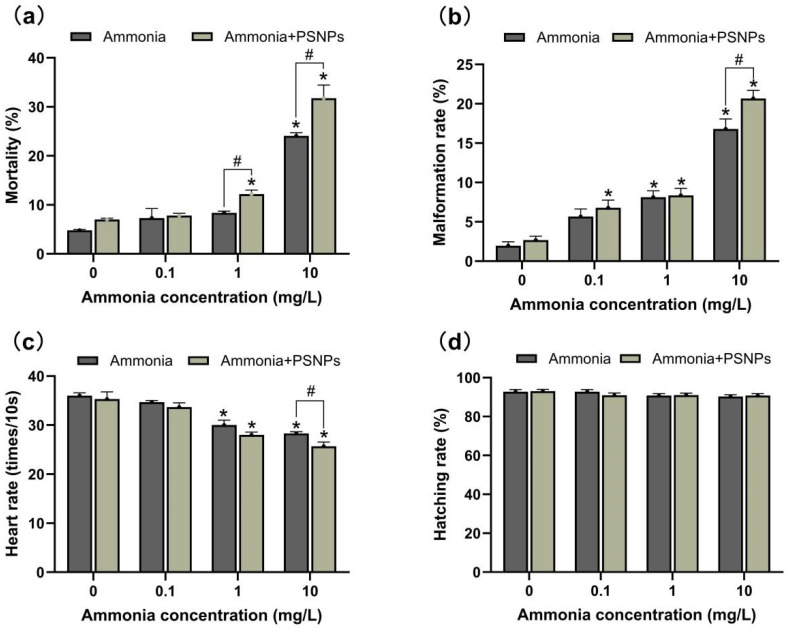
Indicators of developmental endpoints of zebrafish embryos (mean ± SEM, n = 3). (**a**) Mortality rate; (**b**) malformation rate; (**c**) heart rate; (**d**) hatching rate; * indicates a significant difference between the control and exposure groups (*p* < 0.05); # indicates a significant difference between the treatment groups of ammonia and PSNPs + ammonia (*p* < 0.05).

The effects of exposure to ammonia alone and ammonia combined with PSNPs e on zebrafish larval mortality, malformation rate, heart rate, and hatchability were analyzed (Figure 3). The results showed that exposure to PSNPs alone had no effect on zebrafish larval development. There was no significant difference in the mortality rate of zebrafish larvae in all exposure groups before 48 h. From 72 h to 120 h, the mortality rate gradually increased, and was significantly higher (*p* < 0.05) in the 10 mg/L ammonia-alone exposure group as well as in the PSNPs + ammonia (1 mg/L; 10 mg/L) co-exposed groups. In addition, the malformation rate was significantly increased in almost all exposed groups (*p* < 0.05). The heart rate of zebrafish larvae was significantly decreased in both the 1 mg/L ammonia and 10 mg/L ammonia groups (*p* < 0.05). Moreover, PSNPs could exacerbate the effect of ammonia on heart rate. All tested concentrations had no effect on the hatching rate of zebrafish embryos. Collectively, these changes in toxicity endpoints indicate that PSNPs exacerbated the toxicity of ammonia on zebrafish embryo development compared to exposure to ammonia alone.

### 3.4. Locomotor Behavior of Larval Zebrafish

**Figure 4:**
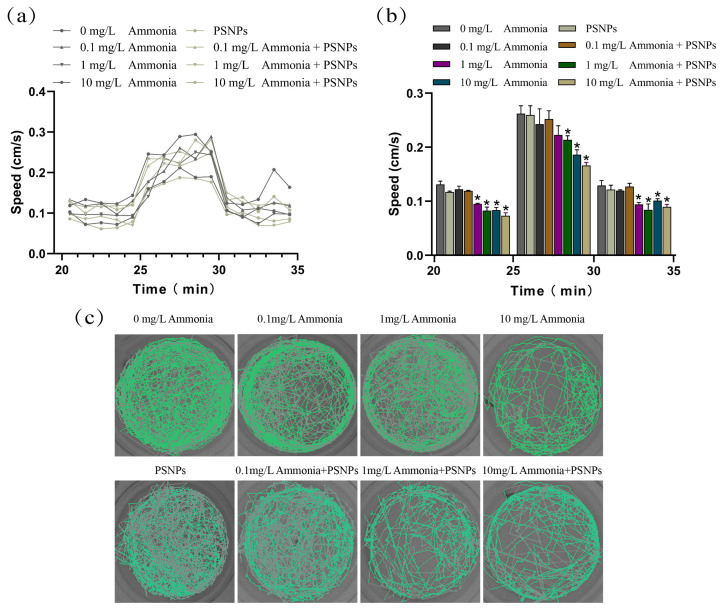
Locomotor behavior of 120 hpf zebrafish larvae (mean ± SEM, n = 3). (**a**) Movement patterns of zebrafish larvae; (**b**) mean velocity (5 min) of zebrafish larvae; (**c**) movement trajectories of zebrafish larvae under dark conditions. * indicates significant difference between control and exposed groups (*p* < 0.05).

The locomotor behavior of zebrafish larvae was measured by the light–dark cycle stimulation test (Figure 4). The test revealed that zebrafish larvae were slower in swimming activity during the light period than during the dark period. Compared to the control zebrafish larvae, zebrafish larvae in these four groups exhibited a delayed decrease in activity during the transition from dark to light, whether exposed to 1 mg/L ammonia or 10 mg/L ammonia, either alone or in combination with PSNPs. Mean larval activity during each photoperiod (5 min) was also quantified (Figure 4b). During the dark period, the mean swimming speed of larvae was significantly lower in the 10 mg/L ammonia-alone exposure group as well as in the PSNPs + ammonia (1 mg/L; 10 mg/L) co-exposed groups (*p* < 0.05). Figure 4c illustrates the trajectory of zebrafish larvae during the photoperiod.

### 3.5. Ammonia Content in Zebrafish Larvae

**Figure 5:**
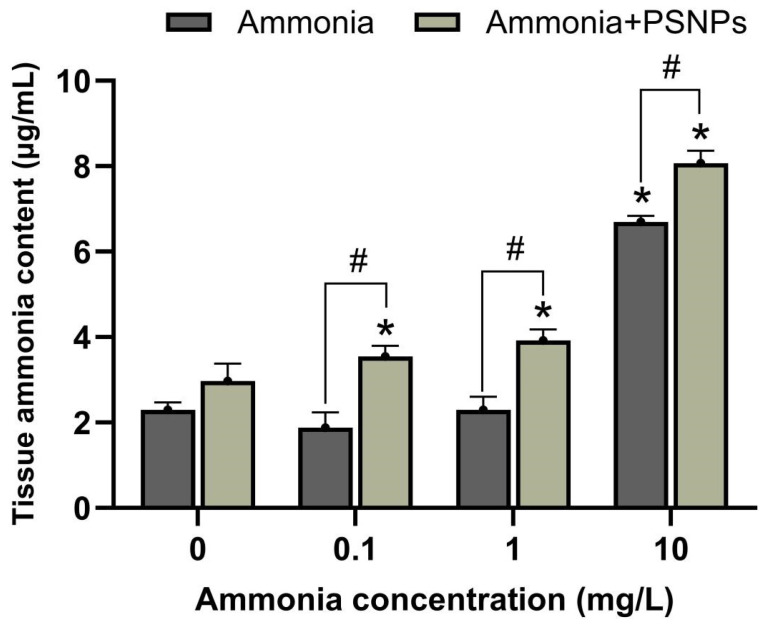
Ammonia content in zebrafish larvae was determined after 120 hpf (mean ± SEM, n = 3). * indicates a significant difference between the control and exposed groups (*p* < 0.05). # indicates a significant difference between the treatment groups of ammonia and PSNPs + ammonia (*p* < 0.05).

The content of ammonia in larval zebrafish was determined (Figure 5). The results showed that the ammonia content was significantly higher in the group exposed to 10 mg/L ammonia (*p* < 0.05). Exposure to PSNPs alone had no significant effect on ammonia levels. However, when ammonia was co-exposed with PSNPs, all co-exposed groups showed a significant elevation in ammonia levels compared to the ammonia-alone exposure groups, indicating that PSNPs could increase ammonia accumulation in zebrafish larvae.

### 3.6. Retinal Tissue Pathological Analysis

**Figure 6:**
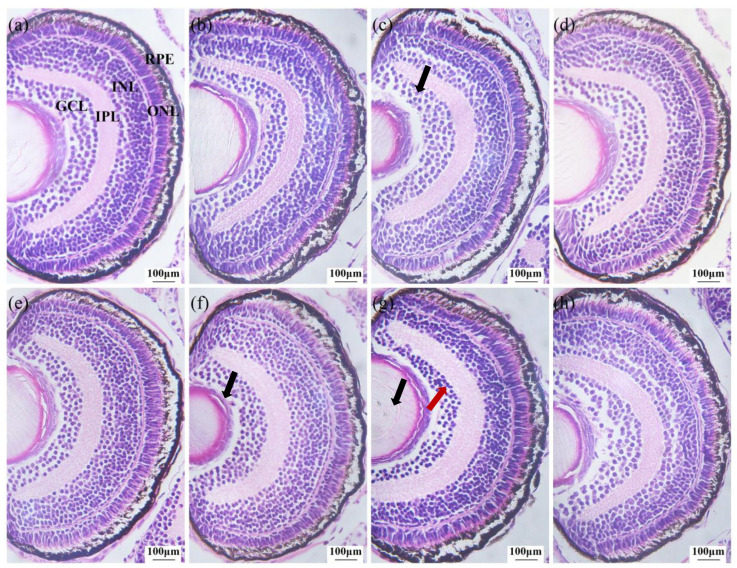
Histopathological analysis of retinal structures in zebrafish larvae. (**a**) Control group; (**b**) 0.1 mg/L ammonia exposure group; (**c**) 1 mg/L ammonia exposure group; (**d**) 10 mg/L ammonia exposure group; (**e**) PSNPs exposure group; (**f**) 0.1 mg/L ammonia + PSNPs exposure group; (**g**) 1 mg/L ammonia + PSNPs exposure group (**h**) 10 mg/L ammonia + PSNPs exposure group. GCL: ganglion cell layer; IPL: inner plexiform layer; INL: inner nuclear layer, ONL: outer nuclear layer; RPE: retinal pigment epithelium (scale bar is 100 μm; n = 10). Black arrows: disorganized nuclei. Red arrow: vacuolization.

To further assess the neurotoxicity of exposure to ammonia alone as well as ammonia co-exposed with PSNPs on zebrafish larvae, larvae eye sections were stained to observe the effects on retinal development (Figure 6). Histological results showed that the nuclear arrangement of the GCL layer cells in the ammonia-alone (10 mg/L) and ammonia (1 mg/L, 10 mg/L) + PSNPs co-exposed groups was disorganized, and the vacuolization of the INL layer was severe in the ammonia (10 mg/L) + PSNPs co-exposed group.

### 3.7. DA Content and AChE Activity

**Figure 7:**
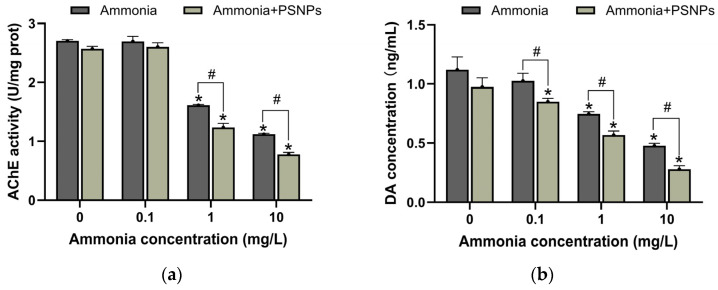
(**a**) Activity of AChE (U/mgprot) and (**b**) DA content (ng/mL) in 120 h zebrafish larvae (mean ± SEM, n = 3). # indicates a significant difference between the treatment groups of ammonia and PSNPs + ammonia (*p* < 0.05), * indicates significant difference between control and exposed groups (*p* < 0.05).

DA content and AChE activity were assayed in zebrafish larvae. Neither DA content nor AChE activity was altered by exposure to PSNPs alone. Exposure to ammonia (1 mg/L and 10 mg/L) significantly decreased the activity of AChE (*p* < 0.05). Co-exposure to PSNPs + ammonia (1 mg/L and 10 mg/L) further decreased the activity of AChE (Figure 7a). DA content significantly decreased in the ammonia (1 mg/L and 10 mg/L)-exposed groups (*p* < 0.05). Moreover, DA content further significantly decreased in all ammonia + PSNPs co-exposed groups (Figure 7b). Consequently, PSNPs can exacerbate this adverse effect of ammonia on DA and AChE activity.

### 3.8. Gene Expression Analysis

**Figure 8:**
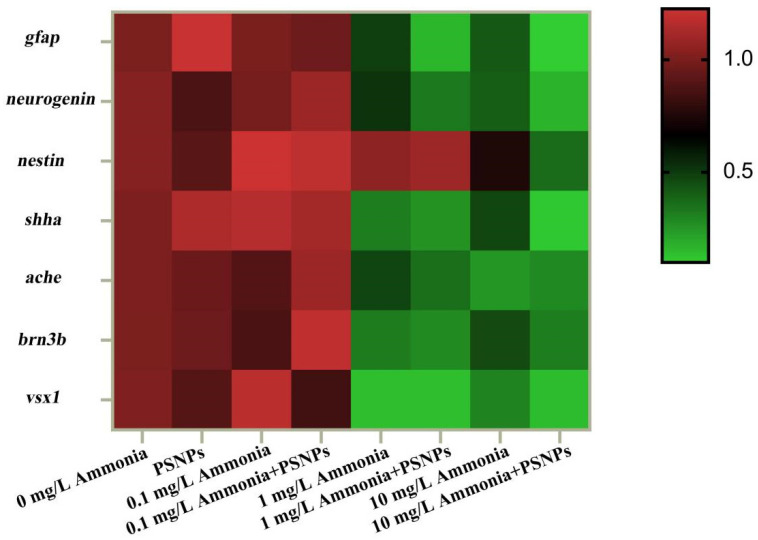
Heatmaps of the expression of neurodevelopment- and retinal development-related genes in zebrafish larvae at 120 hpf (mean ± SEM, n = 3). Values in the heatmaps are derived from log base 2-transformed fold change in gene expression.

The expression levels of neurodevelopment-related genes and retinal development-related genes in zebrafish larvae were assayed at 120 hpf (Figure 8). The neurodevelopment-related genes *gfap*, *neurogenin*, and *shha* showed significant downregulation (*p* < 0.05) in the ammonia-alone (1 mg/L and 10 mg/L) exposure groups, and these genes were further downregulated in the PSNPs + ammonia (1 mg/L and 10 mg/L) co-exposure groups. The *nestin* gene was significantly downregulated (*p* < 0.05) only in the PSNPs + ammonia (10 mg/L) co-exposure group. The *ache* gene showed significant downregulation (*p* < 0.05) in the ammonia-alone (1 mg/L and 10 mg/L) exposure groups as well as in the ammonia (1 mg/L and 10 mg/L) combined with PSNPs exposure groups, and the downregulation was more pronounced with increasing ammonia concentrations. The retinal development-related genes *brn3b* and *vsx1* showed significant downregulation (*p* < 0.05) in the ammonia-alone (1 mg/L and 10 mg/L) exposure groups as well as in their combined exposure group with PSNPs, and these genes were further downregulated in the PSNPs + ammonia (10 mg/L) co-exposure group.

## 4. Discussion

Our results showed that the existence of PSNPs significantly enhanced the accumulation of ammonia in zebrafish larvae compared to the ammonia-alone exposure groups. It was verified PSNPs could act as carriers or absorbents to enhance the enrichment of inorganic pollutants such as Au ions in zebrafish embryos [24] and the accumulation of organic pollutants such as polychlorinated biphenyls in *Daphnia magna* [25], due to their large specific surface area and pore size. In addition, recent studies have also found that PSNPs may impair skin cell function and reduce ion regulation and ammonia excretion in zebrafish embryos, resulting in elevated levels of ammonia in zebrafish [26,27]. Therefore, we speculated that PSNPs may act as carriers of ammonia or influence ammonia metabolism, resulting in significant ammonia enrichment in zebrafish larvae. Taking the serious neurotoxic effects of ammonia on fish into consideration, the joint effects of PSNPs and ammonia should not be ignored.

The locomotor behavior of zebrafish is a sensitive indicator of neurotoxicity induced by various environmental pollutants [28,29,30,31]. It has been proven that locomotor behavior represents the initial response to altered conditions and usually serves as the first line of defense against stress [32]. Accordingly, we examined the locomotor behavior of zebrafish larvae and the results showed that the locomotor activities of zebrafish larvae were reduced in the ammonia-exposed groups and this phenomenon was further exacerbated in the PSNPs + ammonia co-exposure groups. Ammonia, as a potent neurotoxin, can alter the behavior of zebrafish by affecting the release of neurotransmitters, gene expression, and other factors in the central nervous system [33]. In this study, zebrafish larvae were subjected to optimal conditions for the experiments. Therefore, the hypoactivity of zebrafish larvae may be attributed to ammonia-induced neurotoxicity. In addition, we found that exposure to PSNPs alone had no effect on the locomotor behavior of zebrafish larvae. Previous studies have shown that PSNPs (100 μg/L) alone have no toxic effects on zebrafish, yet PSNPs enhance the toxicity of environmental pollutants [19,34], which is consistent with our study. In addition, it has been shown that various environmental pollutants can activate environmental hormetic responses that induce different adaptive reactions [35,36]. In the present study, PSNPs may have also activated environmental hormetic responses to ammonia, enhancing its toxicity. Thus, one possible reason for the further hypoactivity in zebrafish larvae in the co-exposed groups is that PSNPs exacerbate ammonia-induced neurotoxicity. As a conclusion, significant hypoactivity is ascribed to ammonia-induced neurotoxicity and PSNPs can exacerbate ammonia-induced neurotoxicity.

It is recognized that the cholinergic and dopaminergic nervous systems play important roles in neural signaling and motor control [37,38,39]. AChE is an enzyme that plays a key role in the cholinergic system, mainly by rapidly hydrolyzing the neurotransmitter acetylcholine, resulting in the termination of the transmission of nerve impulses by acetylcholine across the synaptic gap [40]. Our results showed that PSNP and ammonia co-exposure exacerbated ammonia-induced inhibition of AChE activity and downregulation of *ache* gene expression. Environmental pollutants, such as organophosphorus, carbamate pesticides, and inorganic pollutants, could inhibit the AChE activity and transcription level of the *ache* gene, thereby leading to a range of neurological dysfunctions [41,42,43,44]. In addition, our results demonstrated that DA content decreased in the ammonia exposure group with concomitant increased mortality and it was further reduced in the PSNPs and ammonia co-exposure groups. Recent studies have shown that environmental pollutants such as trisodium phosphate and polybrominated diphenyl induce neurotoxicity by decreasing systemic dopamine levels and downregulating genes involved in the development of dopaminergic neurons, leading to increased mortality [45,46,47]. Thus, it is reasonable to assume that PSNPs could exacerbate ammonia-induced neurobehavioral changes by decreasing DA secretion. In conclusion, we speculated that altered neurobehavior in zebrafish larvae may be related to abnormalities in the cholinergic system and dopamine neurons, and PSNPs exacerbate the neurotoxic effect triggered by ammonia.

The retina of the zebrafish is also an important part of the nervous system, which is important for converting light signals into neuroelectric signals [48]. Abnormalities in the structure of the retina affect the normal functioning of retinal cells and neural signaling, which causes changes in neurobehavior [49,50]. In this experiment, we found that ammonia exposure disorganized the arrangement of nuclei in the GCL, and the INL showed different vacuolization. Moreover, we found that retinal damage was further exacerbated in the co-exposure group. The proper structure and function of the GCL and INL are essential for the transmission and processing of visual information [51,52]. The biconical cells in the INL are responsible for transmitting light signals received by photoreceptor cells to ganglion cells, which transmit the signals from the retina to neurons in the brain [53]. Therefore, an abnormality in these structures would affect their processing of visual signals. It may also have a wide range of effects on other brain regions and the nervous system through the connectivity and feedback mechanisms of the nervous system, triggering neurotoxic reactions manifested as behavioral abnormalities, reflecting cognitive dysfunction [54]. In addition, we examined the expression of the genes *brn3b* and *vsx1*, which are associated with the development of GCL and INL. The *brn3b* and *vsx1* genes play important roles in the development and function of the GCL and INL of the retina, respectively [55,56]. In our study, *brn3b* and *vsx1* were significantly downregulated, which is consistent with structural abnormalities in the GCL and the INL. Based on the above statement, we speculate that abnormalities in the INL and GCL are important factors in the neurobehavioral alterations in zebrafish larvae.

Considering that changes in neurobehavior during the early life stages of zebrafish are linked to the development and differentiation of the nervous system, we also analyzed the expression of genes related to the development of the nervous system as well as differentiation (*gfap*, *neurogenin*, *nestin*, and *shha*). Our results showed that the expression levels of these genes were significantly downregulated, and the changes were exacerbated in the co-exposed groups. Downregulation of the expression of *neurogenin* and *shha* genes could impede neuronal proliferation and differentiation and affect neuronal cell numbers, eventually leading to abnormal neurobehavior [57,58,59]. The *nestin* gene is a marker for neuronal and precursor cells, and its decreased expression may indicate a reduction in the number of neuronal cells [60]. It has been shown that an insufficient or reduced number of neuronal cells may lead to problems in sensory perception or motor coordination [61]. As a case in point, a reduction in the number of neuronal cells may weaken the zebrafish’s perception of changes in water flow, affecting its ability to maintain balance in the water and swim normally [62]. In addition, downregulation of *gfap* gene expression may imply damage to astrocytes, which may lead to decreased neuronal cell stability [63]. Based on the above results, it was reasonable to conclude that ammonia-induced neurotoxicity in zebrafish larvae may be associated with abnormalities in neuronal cells and PSNPs can exacerbate ammonia-induced neurotoxic effect.

## 5. Conclusions

PSNPs significantly increase ammonia accumulation in zebrafish and ultimately exacerbate ammonia-induced neurotoxicity. The mechanism by which ammonia or ammonia combined with PSNPs induces neurotoxicity may be related to alterations in the structure of the zebrafish retina, disturbances in the cholinergic system and dopaminergic neurons, and abnormalities in the expression of related genes. However, there are still some limitations in this study. For example, the issue of how differences in the physicochemical properties of PSNPs themselves affect their ammonia adsorption and the combined toxicity of PSNPs and ammonia has not been deeply studied either. Future research should focus on in-depth explanation of the molecular mechanisms of the synergistic effects of ammonia and PSNPs, exploring effective environmental monitoring and management strategies to reduce the potential risks of the two environmental pollutants to the aquatic ecosystem and human health.

## Figures and Tables

**Table 1:** Primer sequences for the genes.

Gene Name	Primer Sequences (from 5′ to 3′)	Gene Bank Accession No.
*ache*	Forward: CCCTCCAGTGGGTACAAGAA	NM_131846
	Reverse: GGGCCTCATCAAAGGTAACA	
*neurogenin*	Forward: CTCCCAGCCCACCAATAAGG	AF024535
	Reverse: CATCCGTGTGCGAAAAGCAG	
*gfap*	Forward: GGATGCAGCCAATCGTAAT	NM_131373
	Reverse: TTCCAGGTCACAGGTCAG	
*shha*	Forward: GCAAGATAACGCGCAATTCGGAGA	DRU30711
	Reverse: ATGCTGGAGAAACATGCCATGCAG	
*nestin*	Forward: ATGCTGGAGAAACATGCCATGCAG	XM_001919887
	Reverse: AGGGTGTTTACTTGGGCCTGAAGA	
*brn3b*	Forward: CATTCCTCGTCCTCCTCCTCTACTC	NC_007112.7
	Reverse: CTGATCGTGCTGCTGTGTCTGG	
*vsx1*	Forward: GAACTTCAGCCGCACCAGTCAG	NC_007128.7
	Reverse: CACAGAAACCCCAAGCCCAAGG	

## Data Availability

The datasets used and/or analyzed during the current study are available from the corresponding author upon reasonable request.

## Supplementary Materials

The following are available online at https://www.mdpi.com/article/10.3390/toxics12120853/s1, Figure S1. TEM image of PSNPs. Scale bars, 500 nm.

## References

[1] Dong Y., Yuan H., Zhang R., Zhu N. (2019). Removal of Ammonia Nitrogen from Wastewater: A Review. Trans. ASABE.

[2] Anderson N., Strader R., Davidson C. (2003). Airborne reduced nitrogen: Ammonia emissions from agriculture and other sources. Environ. Int..

[3] Cheng C.-H., Ma H.-L., Su Y.-L., Deng Y.-Q., Feng J., Xie J.-W., Chen X.-L., Guo Z.-X. (2019). Ammonia toxicity in the mud crab (*Scylla paramamosain*): The mechanistic insight from physiology to transcriptome analysis. Ecotoxicol. Environ. Saf..

[4] Schneider M. (1996). The importance of ammonia in mammalian cell culture. J. Biotechnol..

[5] Egnew N., Renukdas N., Ramena Y., Yadav A.K., Kelly A.M., Lochmann R.T., Sinha A.K. (2019). Physiological insights into largemouth bass (*Micropterus salmoides*) survival during long-term exposure to high environmental ammonia. Aquat. Toxicol..

[6] Wang X., Li J., Chen J., Cui L., Li W., Gao X., Liu Z. (2020). Water quality criteria of total ammonia nitrogen (TAN) and un-ionized ammonia (NH_3_-N) and their ecological risk in the Liao River, China. Chemosphere.

[7] Cong M., Wu H., Yang H., Zhao J., Lv J. (2017). Gill damage and neurotoxicity of ammonia nitrogen on the clam Ruditapes philippinarum. Ecotoxicology.

[8] Lin L.-Y., Cheng C.-A., Liu S.-T., Horng J.-L. (2024). Investigation of ammonia-induced lethal toxicity toward ion regulation in zebrafish embryos. Comp. Biochem. Physiol. Part C Toxicol. Pharmacol..

[9] Ip Y. (2010). Ammonia production, excretion, toxicity, and defense in fish: A review. Front. Physiol..

[10] Randall D.J., Tsui T.K.N. (2002). Ammonia toxicity in fish. Mar. Pollut. Bull..

[11] Moore C.J. (2008). Synthetic polymers in the marine environment: A rapidly increasing, long-term threat. Environ. Res..

[12] Cole M., Lindeque P., Halsband C., Galloway T.S. (2011). Microplastics as contaminants in the marine environment: A review. Mar. Pollut. Bull..

[13] Li W.C., Tse H.F., Fok L. (2016). Plastic waste in the marine environment: A review of sources, occurrence and effects. Sci. Total Environ..

[14] Wagner M., Scherer C., Alvarez-Muñoz D., Brennholt N., Bourrain X., Buchinger S., Fries E., Grosbois C., Klasmeier J., Marti T. (2014). Microplastics in freshwater ecosystems: What we know and what we need to know. Environ. Sci. Eur..

[15] Yu F., Yang C., Zhu Z., Bai X., Ma J. (2019). Adsorption behavior of organic pollutants and metals on micro/nanoplastics in the aquatic environment. Sci. Total Environ..

[16] Brennecke D., Duarte B., Paiva F., Caçador I., Canning-Clode J. (2016). Microplastics as vector for heavy metal contamination from the marine environment. Estuar. Coast. Shelf Sci..

[17] Zhao M., Huang L., Arulmani S.R.B., Yan J., Wu L., Wu T., Zhang H., Xiao T. (2022). Adsorption of Different Pollutants by Using Microplastic with Different Influencing Factors and Mechanisms in Wastewater: A Review. Nanomaterials.

[18] Chen Q., Yin D., Jia Y., Schiwy S., Legradi J., Yang S., Hollert H. (2017). Enhanced uptake of BPA in the presence of nanoplastics can lead to neurotoxic effects in adult zebrafish. Sci. Total Environ..

[19] Zuo J., Huo T., Du X., Yang Q., Wu Q., Shen J., Liu C., Hung T.-C., Yan W., Li G. (2021). The joint effect of parental exposure to microcystin-LR and polystyrene nanoplastics on the growth of zebrafish offspring. J. Hazard. Mater..

[20] Kuang Y., Guo H., Ouyang K., Wang X., Li D., Li L. (2023). Nano-TiO_2_ aggravates immunotoxic effects of chronic ammonia stress in zebrafish (*Danio rerio*) intestine. Comp. Biochem. Physiol. Part C Toxicol. Pharmacol..

[21] Torres-Ruiz M., De la Vieja A., de Alba Gonzalez M., Esteban Lopez M., Castaño Calvo A., Cañas Portilla A.I. (2021). Toxicity of nanoplastics for zebrafish embryos, what we know and where to go next. Sci. Total Environ..

[22] Rinkwitz S., Mourrain P., Becker T.S. (2011). Zebrafish: An integrative system for neurogenomics and neurosciences. Prog. Neurobiol..

[23] Froehlicher M., Liedtke A., Groh K.J., Neuhauss S.C.F., Segner H., Eggen R.I.L. (2009). Zebrafish (*Danio rerio*) neuromast: Promising biological endpoint linking developmental and toxicological studies. Aquat. Toxicol..

[24] Lee W.S., Cho H.-J., Kim E., Huh Y.H., Kim H.-J., Kim B., Kang T., Lee J.-S., Jeong J. (2019). Correction: Bioaccumulation of polystyrene nanoplastics and their effect on the toxicity of Au ions in zebrafish embryos. Nanoscale.

[25] Jiang R., Lin W., Wu J., Xiong Y., Zhu F., Bao L.-J., You J., Ouyang G., Zeng E.Y. (2018). Quantifying nanoplastic-bound chemicals accumulated in *Daphnia magna* with a passive dosing method. Environ. Sci. Nano.

[26] Zhao Y., Bao Z., Wan Z., Fu Z., Jin Y. (2020). Polystyrene microplastic exposure disturbs hepatic glycolipid metabolism at the physiological, biochemical, and transcriptomic levels in adult zebrafish. Sci. Total Environ..

[27] Rehman A., Huang F., Zhang Z., Habumugisha T., Yan C., Shaheen U., Zhang X. (2024). Nanoplastic contamination: Impact on zebrafish liver metabolism and implications for aquatic environmental health. Environ. Int..

[28] Poopal R.-K., He Y., Zhao R., Li B., Ramesh M., Ren Z. (2021). Organophosphorus-based chemical additives induced behavioral changes in zebrafish (*Danio rerio*): Swimming activity is a sensitive stress indicator. Neurotoxicol. Teratol..

[29] Chen X., Huang C., Wang X., Chen J., Bai C., Chen Y., Chen X., Dong Q., Yang D. (2012). BDE-47 disrupts axonal growth and motor behavior in developing zebrafish. Aquat. Toxicol..

[30] Zhu W., Liu Y., Xuan X., Xu Z., Gao P., Jin Z., Hong H., Sun H. (2024). Dihalogenated nitrophenols exposure induces developmental neurotoxicity in zebrafish embryo. Ecotoxicol. Environ. Saf..

[31] Tilton F.A., Bammler T.K., Gallagher E.P. (2011). Swimming impairment and acetylcholinesterase inhibition in zebrafish exposed to copper or chlorpyrifos separately, or as mixtures. Comp. Biochem. Physiol. Part C Toxicol. Pharmacol..

[32] Wong B.B.M., Candolin U. (2014). Behavioral responses to changing environments. Behav. Ecol..

[33] Lin L.-Y., Horng J.-L., Cheng C.-A., Chang C.-Y., Cherng B.-W., Liu S.-T., Chou M.-Y. (2022). Sublethal ammonia induces alterations of emotions, cognition, and social behaviors in zebrafish (*Danio rerio*). Ecotoxicol. Environ. Saf..

[34] Ling X., Zuo J., Pan M., Nie H., Shen J., Yang Q., Hung T.-C., Li G. (2022). The presence of polystyrene nanoplastics enhances the MCLR uptake in zebrafish leading to the exacerbation of oxidative liver damage. Sci. Total Environ..

[35] Calabrese E.J. (2008). Hormesis: Why it is important to toxicology and toxicologists. Environ. Toxicol. Chem..

[36] Scuto M., Rampulla F., Reali G.M., Spanò S.M., Trovato Salinaro A., Calabrese V. (2024). Hormetic Nutrition and Redox Regulation in Gut–Brain Axis Disorders. Antioxidants.

[37] Halder N., Lal G. (2021). Cholinergic System and Its Therapeutic Importance in Inflammation and Autoimmunity. Front. Immunol..

[38] Takahashi T. (2021). Multiple Roles for Cholinergic Signaling from the Perspective of Stem Cell Function. Int. J. Mol. Sci..

[39] Schultz W. (2007). Multiple Dopamine Functions at Different Time Courses. Annu. Rev. Neurosci..

[40] Silman I., Sussman J.L. (2008). Acetylcholinesterase: How is structure related to function?. Chem. Biol. Interact..

[41] Fu H., Xia Y., Chen Y., Xu T., Xu L., Guo Z., Xu H., Xie H.Q., Zhao B. (2018). Acetylcholinesterase Is a Potential Biomarker for a Broad Spectrum of Organic Environmental Pollutants. Environ. Sci. Technol..

[42] Del Pino J., Zeballos G., Anadon M.J., Capo M.A., Díaz M.J., García J., Frejo M.T. (2014). Higher sensitivity to cadmium induced cell death of basal forebrain cholinergic neurons: A cholinesterase dependent mechanism. Toxicology.

[43] Lionetto M.G., Caricato R., Calisi A., Giordano M.E., Schettino T. (2013). Acetylcholinesterase as a Biomarker in Environmental and Occupational Medicine: New Insights and Future Perspectives. BioMed Res. Int..

[44] Richetti S.K., Rosemberg D.B., Ventura-Lima J., Monserrat J.M., Bogo M.R., Bonan C.D. (2011). Acetylcholinesterase activity and antioxidant capacity of zebrafish brain is altered by heavy metal exposure. NeuroToxicology.

[45] Ren X., Liu Z., Zhang R., Shao Y., Duan X., Sun B., Zhao X. (2024). Nanoplastics aggravated TDCIPP-induced transgenerational developmental neurotoxicity in zebrafish depending on the involvement of the dopamine signaling pathway. Environ. Toxicol. Pharmacol..

[46] Mahapatra A., Gupta P., Suman A., Ray S.S., Malafaia G., Singh R.K. (2023). Unraveling the mechanisms of perfluorooctanesulfonic acid-induced dopaminergic neurotoxicity and microglial activation in developing zebrafish. Sci. Total Environ..

[47] Wang Q., Lam J.C.-W., Man Y.-C., Lai N.L.-S., Kwok K.Y., Guo Y.y., Lam P.K.-S., Zhou B. (2015). Bioconcentration, metabolism and neurotoxicity of the organophorous flame retardant 1,3-dichloro 2-propyl phosphate (TDCPP) to zebrafish. Aquat. Toxicol..

[48] Bollmann J.H. (2019). The Zebrafish Visual System: From Circuits to Behavior. Annu. Rev. Vis. Sci..

[49] Engert F., Portugues R. (2011). Adaptive Locomotor Behavior in Larval Zebrafish. Front. Syst. Neurosci..

[50] Chen L., Huang Y., Huang C., Hu B., Hu C., Zhou B. (2013). Acute exposure to DE-71 causes alterations in visual behavior in zebrafish larvae. Environ. Toxicol. Chem..

[51] Icha J., Kunath C., Rocha-Martins M., Norden C. (2016). Independent modes of ganglion cell translocation ensure correct lamination of the zebrafish retina. J. Cell Biol..

[52] Linden R., Zhang Y., Yang Y., Trujillo C., Zhong W., Leung Y.F. (2012). The Expression of irx7 in the Inner Nuclear Layer of Zebrafish Retina Is Essential for a Proper Retinal Development and Lamination. PLoS ONE.

[53] Shi Q., Wang Z., Chen L., Fu J., Han J., Hu B., Zhou B. (2019). Optical toxicity of triphenyl phosphate in zebrafish larvae. Aquat. Toxicol..

[54] Nishimura Y., Murakami S., Ashikawa Y., Sasagawa S., Umemoto N., Shimada Y., Tanaka T. (2015). Zebrafish as a systems toxicology model for developmental neurotoxicity testing. Congenit. Anom..

[55] de Melo J., Qiu X., Du G., Cristante L., Eisenstat D.D. (2003). Dlx1, Dlx2, Pax6, Brn3b, and Chx10 homeobox gene expression defines the retinal ganglion and inner nuclear layers of the developing and adult mouse retina. J. Comp. Neurol..

[56] Ohtoshi A., Wang S.W., Maeda H., Saszik S.M., Frishman L.J., Klein W.H., Behringer R.R. (2004). Regulation of Retinal Cone Bipolar Cell Differentiation and Photopic Vision by the CVC Homeobox Gene Vsx1. Curr. Biol..

[57] Hulme A.J., Maksour S., St-Clair Glover M., Miellet S., Dottori M. (2022). Making neurons, made easy: The use of Neurogenin-2 in neuronal differentiation. Stem Cell Rep..

[58] Seo S., Lim J.-W., Yellajoshyula D., Chang L.-W., Kroll K.L. (2007). Neurogenin and NeuroD direct transcriptional targets and their regulatory enhancers. EMBO J..

[59] Palma V.n., Lim D.A., Dahmane N., Sánchez P., Brionne T.C., Herzberg C.D., Gitton Y., Carleton A., Álvarez-Buylla A., Altaba A.R.i. (2005). Sonic hedgehog controls stem cell behavior in the postnatal and adult brain. Development.

[60] Bernal A., Arranz L. (2018). Nestin-expressing progenitor cells: Function, identity and therapeutic implications. Cell. Mol. Life Sci..

[61] Chandrasekhar A., Guo S., Masai I., Nicolson T., Wu C.-F. (2017). Zebrafish: From genes and neurons to circuits, behavior and disease. J. Neurogenet..

[62] Son J.-H., Gerenza A.K., Bingener G.M., Bonkowsky J.L. (2022). Hypoplasia of dopaminergic neurons by hypoxia-induced neurotoxicity is associated with disrupted swimming development of larval zebrafish. Front. Cell. Neurosci..

[63] Brenner M., Messing A. (2021). Regulation of GFAP Expression. ASN Neuro.

