# Supplementary material for: Polystyrene Nanomicroplastics Aggravate Ammonia-Induced Neurotoxic Effects in Zebrafish Embryos
Source: Toxics. 2024 Nov 26;12(12):853. doi: 10.3390/toxics12120853 (PMC11679152; doi:10.3390/toxics12120853)
Supplement: Supplementary file 1 [file toxics-12-00853-s001.zip › toxics-3278162-supplementary.pdf]

Supplementary Materials

Polystyrene Nanomicroplastics Aggravate Ammonia-Induced Neurotoxic Effects in Zebrafish Embryos

Dan Xing [1,2,†], Wenting Zheng [1,†], Huiming Zhou [3], Guangyu Li [1], Yan Li [1], Jingwen Jia [1], Haoling Liu [1], Ning Luan [1] and Xiaolin Liu *

[1] College of Fisheries, Huazhong Agricultural University, Wuhan 430070, China; dan.xing@ceic.com (D.X.); zheng_teiko@hotmail.com (W.Z.); liguangyu@mail.hzau.edu.cn (G.L.); m15623588901@163.com (Y.L.); 13720270799@163.com (J.J.); liuhaoling@webmail.hzau.edu.cn (H.L.); luanning@webmail.hzau.edu.cn (N.L.)

[2] CHN Energy Dadu River Hydropower Development Co., Ltd., Chengdu 610000, China

[3] Jiangxi Fisheries Research Institute, Nanchang 330039, China; mhz_507@webmail.hzau.edu.cn

* Correspondence: liuxiaolin@webmail.hzau.edu.cn

† These authors contributed equally to this work.

This Supporting Information contains:

Characterization of higher precision PSNPs in distilled water. **Figure S1.**

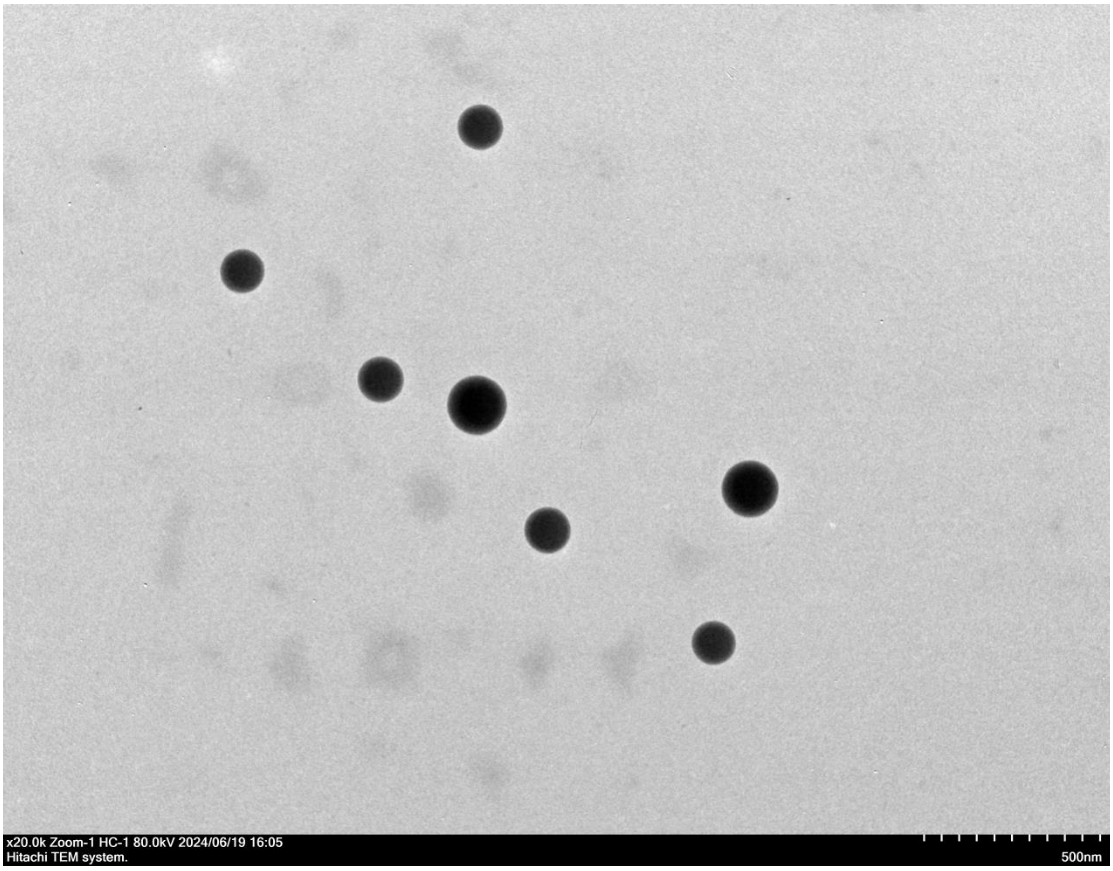

Figure S1. TEM image of PSNPs. Scale bars, 500 nm.
